# Scaling biodiversity-stability relationships from populations to meta-communities across trophic levels

**DOI:** 10.1038/s41467-026-75366-1

**Published:** 2026-07-07

**Authors:** Ming-Qiang Wang, Shaopeng Wang, Xiaojuan Liu, Lei Zhao, Douglas Chesters, Helge Bruelheide, Yi Li, Jing-Ting Chen, Shan Li, Qing-Song Zhou, Keping Ma, Arong Luo, Andreas Schuldt, Chao-Dong Zhu, Georg Albert

**Affiliations:** 1 Mountain Ecological Restoration and Biodiversity Conservation Key Laboratory of Sichuan Province, Chengdu Institute of Biology, Chinese Academy of Sciences, Chengdu, China; 2 China-Croatia Belt and Road Joint Laboratory on Biodiversity and Ecosystem Services, Chengdu Institute of Biology, Chinese Academy of Sciences, Chengdu, China; 3 State Key Laboratory of Animal Biodiversity Conservation and Integrated Pest Management, Institute of Zoology, Chinese Academy of Sciences, Beijing, China; 4 Department of Forest Nature Conservation, University of Göttingen, Göttingen, Germany; 5 Institute of Ecology, College of Urban and Environmental Science, and Key Laboratory for Earth Surface Processes of the Ministry of Education, Peking University, Beijing, China; 6 State Key Laboratory of Vegetation and Environmental Change, Institute of Botany, Chinese Academy of Sciences, Beijing, China; 7 Beijing Key Laboratory of Biodiversity and Organic Farming, College of Resources and Environmental Sciences, China Agricultural University, Beijing, China; 8 College of Biological Sciences/International College, University of Chinese Academy of Sciences, Beijing, China; 9 Institute of Biology/Geobotany and Botanical Garden, Martin Luther University Halle-Wittenberg, Halle (Saale), Germany; 10 German Centre for Integrative Biodiversity Research (iDiv) Halle-Jena-Leipzig, Leipzig, Germany

**Keywords:** Forest ecology, Population dynamics, Community ecology

## Abstract

Ecological stability is essential for maintaining ecosystem functioning, but may be imperiled by biodiversity loss. Although the scaling of diversity-stability relationships from populations to communities and metacommunities has been studied within single trophic levels, it remains poorly understood when considering interactions between trophic levels. Here, we utilize data collected from a large-scale forest biodiversity experiment to investigate the scaling of temporal stability from populations, to communities, and meta-communities in a plant-herbivore system, allowing us to disentangle the relative role of top-down and bottom-up regulation. We observe that biodiversity has generally stabilizing effects within and between trophic levels. Specifically, species diversity of herbivores shows strong stabilizing top-down effects by enhancing species stability and asynchrony of plants that cascade to higher levels of organization. In contrast, bottom-up effects play a much smaller role. Our study therefore highlights the importance of top-down processes in safeguarding plant stability across levels of organization, while simultaneously providing a framework that allows the investigation of the multi-layered nature of stability mechanisms that needs to be considered for a successful and sustainable ecosystem management.

## Abstract (web summary)

Biodiversity can stabilize ecosystems, but how stability scales across trophic levels is unclear. Using a forest biodiversity experiment, this study shows that herbivore diversity stabilizes plant populations, communities, and metacommunities via top-down effects.

## Introduction

Biodiversity is an important component for buffering the negative effects of environmental fluctuations on species dynamics and associated ecosystem functions and services crucial for human well-being (i.e., insurance hypothesis^1^). In times of global environmental change, this buffering is becoming increasingly important, as environmental fluctuations are expected to increase due to anthropogenic pressure^2,3^ and climate change^4,5^. Hence, global biodiversity loss undermines the ability of ecosystems to mitigate environmental fluctuations and thereby threatens the stability of ecosystem functions and services^6^. Since the stability of ecosystem functions is rooted in the stability of ecological communities (e.g., productivity of plant communities^7,8^), understanding the potential drivers of the stability of ecological communities has become a key scientific challenge. However, existing studies mainly focus on the stability of single trophic levels, mostly plants^9–11^ (but see Klink et al.^12^). They thus fail to capture top-down and bottom-up processes that link species across trophic levels and that are expected to play important roles for stability^13,14^, severely limiting our ability to capture the mechanisms underlying this stability.

In addition to biodiversity loss, increasing reports of biotic homogenization at the landscape scale^15–17^ have raised concerns about how community stability affects the stability of meta-communities^18,19^. A theoretical framework introduced by Wang and Loreau^18^ allows us to address this issue. It shows that, just as the stability of species can affect community stability (alpha invariability *sensu* Wang and Loreau^18^), the stability of communities can drive meta-community stability (gamma invariability *sensu* Wang and Loreau^18^). Similarly, complementary temporal dynamics among species populations (i.e., species asynchrony) or communities (i.e., community asynchrony; beta variability *sensu* Wang and Loreau^18^) can promote community and meta-community stability, respectively. In light of this framework, a number of theoretical and empirical studies have demonstrated the relationship between species diversity and ecosystem stability across multiple scales. These studies suggest that for plant communities, species diversity can drive meta-community stability through altering species stability, species and community asynchrony, as well as beta diversity (i.e., diversity turnover between communities)^15,20,21^. These effects can be further modified by trophic interactions, such as grazing^21^.

It has long been recognized that top-down and bottom-up mechanisms affect communities simultaneously^22–26^. In plant–herbivore systems, plants provide resources to herbivores, thus fundamentally constraining herbivore abundances^27,28^. At the same time, herbivore feeding pressure can limit plant growth^29,30^ and reproductive success^31,32^, which in turn affects herbivore community dynamics^33^. This bi-directional interaction spawned co-evolutionary interactions that play an integral role in creating and maintaining biodiversity^34,35^, providing communities and ecosystems with greater resilience and adaptability in the face of environmental changes^36^. Therefore, understanding and studying these bi-directional interactions and co-evolutionary processes is of great importance also for biodiversity conservation. However, the cyclic nature of top-down and bottom-up processes renders their simultaneous quantification a major challenge in ecological research that focuses on the interaction between multiple trophic levels^37^. The issue is further complexified by a mismatch in the operational scale of plants and herbivores, with plants acting locally whereas herbivores are more mobile and thereby integrate effects at larger spatial scales^38^. As a result, identifying whether the stability in plant–herbivore interactions, and food webs in general, is predominantly top-down or bottom-up controlled requires a multi-scale approach.

There are three potential entry points through which adjacent trophic levels could affect each other’s stability, mirroring within-trophic-level relationships^19,39^. First, the diversity of one trophic level affects the stability of the adjacent trophic level. For example, plant diversity enhances plant productivity^40,41^, which stabilizes generalized herbivore community dynamics while simultaneously destabilizing specialist species populations^33^. The diversity of plant species, especially when experimentally manipulated, can also promote the diversity of herbivore communities^27,42^, which enhances the stability of the herbivore (meta-)community^12^. In contrast, we still lack understanding of the roles of herbivore diversity in moderating plant community dynamics (but see ref. ^43^). Second, asynchronous dynamics of one trophic level stabilize its impact and thereby streamline the effect on the dynamics of the adjacent trophic level. This pathway may play a particularly important role in generalized feeding interactions. A (meta-)community composed of generalist herbivores that utilize a wide range of hosts could dynamically adapt to asynchronous dynamics of plant productivity^44^. Similarly, asynchronous dynamics of herbivore abundances apply a constant top-down control^45^ that could alter the stability of the plant (meta-)community productivity. Third, the stability of a given organizational level (i.e., population or community) could determine the stability at a higher level of organization (i.e., community or meta-community) and adjacent trophic level. In contrast to the asynchrony pathway, this should benefit all feeding interactions regardless of being specialized or generalized. While the three pathways likely act in concert, it remains unclear if (1) they stabilize or destabilize adjacent trophic levels, (2) single pathways dominate over others, and (3) the contribution of top-down and bottom-up processes to ecosystem dynamics is similar in sign and magnitude.

**Fig. 1:**
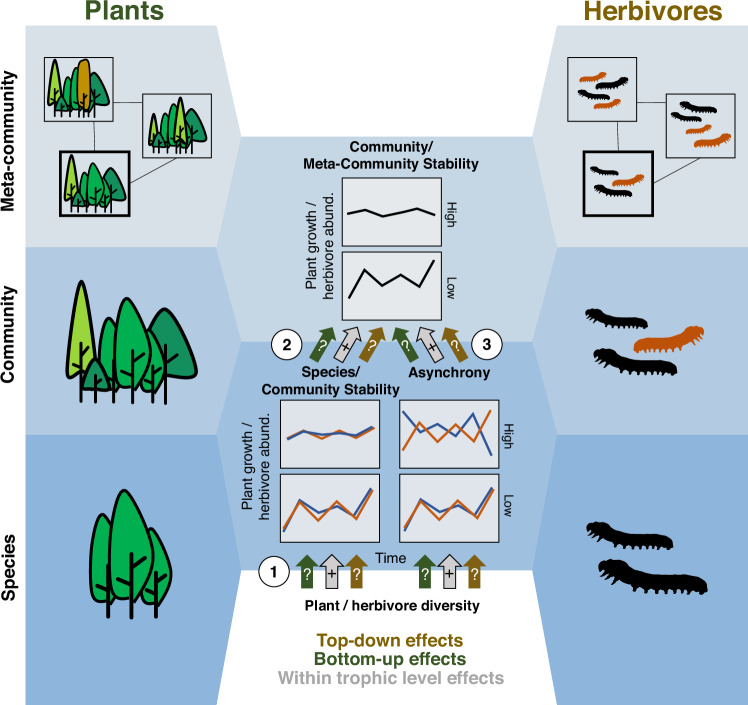
Conceptual figure illustrating the processes determining stability across levels of organization within and across trophic levels. (1) Diversity affects asynchrony or stability of species and communities (depicted as colored lines in the lower panels), (2) stability (bottom left panels) or (3) asynchrony (bottom right panels) of species and communities then enhance community and meta-community stability (black lines in upper panels). The cascading effects are expected to be positive within each trophic level (gray arrows), but bottom-up (green arrows) and top-down (brown arrows) effects between trophic levels remain untested.

To disentangle the top-down and bottom-up regulations of temporal stability in a plant-herbivore system, we utilize the framework introduced by Wang and Loreau^18^ that connects effects across levels of organizations (i.e., populations, communities, meta-communities). This allows us to account for the differences in the operational scale of plants and herbivores while quantifying and comparing the strength of top-down and bottom-up processes simultaneously. Specifically, we use time-series data (2017–2022) of lepidopteran larvae and tree growth collected from a large-scale tree biodiversity experiment in subtropical China, the BEF China experiment^46^. Utilizing a resampling approach, we create 1000 meta-communities, which enable us to partition stability, asynchrony, and diversity effects across multiple levels of organization. We expect that stability metrics of one trophic level are affected by diversity, asynchrony, and stability of the adjacent trophic level (Figs. 1 and S1). Because plant diversity was experimentally manipulated^46^, we expect that bottom-up processes are more pronounced than top-down processes and that tree diversity shows particularly strong effects. However, whether diversity effects predominantly alter stability relationships within or across trophic levels is unclear. Hence, diversity, stability, and asynchrony pathways could all mediate top-down and bottom-up effects. We therefore set out to test (1) which pathways dominate top-down and bottom-up effects and (2) whether they stabilize or destabilize adjacent trophic levels. This allows us to generate insights into the drivers of stability, how they scale across levels of organization (i.e., from populations to meta-communities), and—most importantly —how they cascade across trophic levels.

## Results

Of the 243 MOTUs included in the final analysis, 132 could be assigned to Lepidoptera families. These family-level identifiable MOTUs were dominated by several species-rich and abundant groups (Table S1), particularly Erebidae (*n* = 40 MOTUs), Geometridae (*n* = 37), and Noctuidae (*n* = 10), which together contributed the largest share of both richness (*n* = 87) and individuals (*n* = 7887). On average, MOTUs exhibited moderately broad generality (mean host breadth = 9.42), indicating that broad-feeding taxa were prevalent in this system. Most abundant MOTUs occurred repeatedly across multiple years, suggesting the herbivore community is structured by persistent generalists rather than specialized taxa.

### Mechanisms linking diversity and stability within trophic levels

**Fig. 2:**
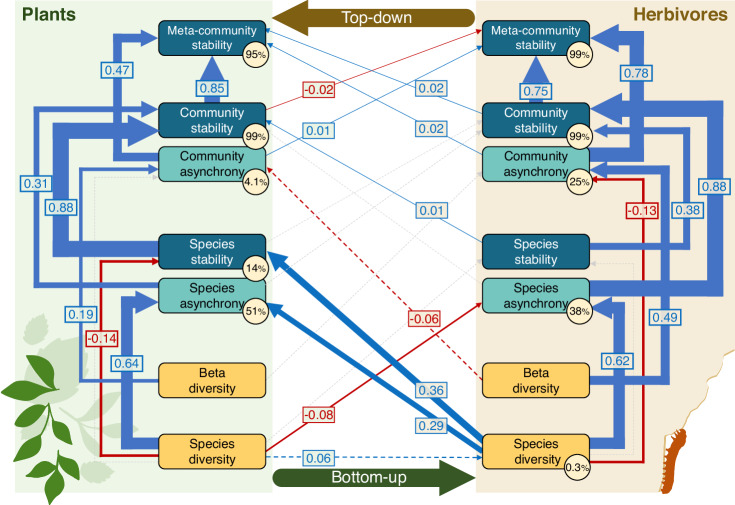
Pathways of stability determining processes within and across trophic levels based on structural equation model results. Direct and indirect pathways through which diversity, asynchrony, and stability at multiple levels of organization determine meta-community stability within trophic levels for plants (left) and herbivores (right), and between trophic levels (middle). Solid blue arrows indicate significant positive effects (*P* ≤ 0.05), solid red arrows show significant negative effects (*P* ≤ 0.05), dashed blue/red arrows indicate marginally significant effects (0.05 < *P* ≤ 0.1), and dashed gray arrows represent nonsignificant coefficients (*P* > 0.1). Arrow width was scaled by the standardized path coefficients. Percentages next to endogenous variables indicate the variance explained by the model (*R*^2^). Model fit statistics: *χ*² = 327.55, DF = 55, *P* < 0.001, CFI = 0.988, RMSEA = 0.070, SRMR = 0.076. For an overview of the statistics, including covariances, see Table S2. Statistical tests were two-sided, and no adjustments were made for multiple comparisons. Source data are provided as a Source Data file.

We observed strong correlations among diversity, asynchrony, and stability across organizational scales within trophic levels. For both plants and herbivores, our structural equation models showed that diversity affected stability largely via modifying asynchrony, both at community (species asynchrony) and meta-community (community asynchrony) levels (Fig. 2; Table S2). While most of these effects were positive, species diversity of herbivores had negative effects on herbivore community asynchrony, reversing some of its benefits. Beta diversity of plants and herbivores consistently promoted community asynchrony. Species stability played a particularly important role in the community stability of plants, but was negatively affected by plant species diversity. Herbivore species stability showed weaker effects and was independent of herbivore species diversity. Meta-community stability was tightly associated with community asynchrony and community stability. While the diversity effects of plants and herbivores were largely similar, asynchrony played a more pronounced role for herbivore stability across organizational scales than for plants, where the stability of the lower organizational levels played a bigger role. Model results were robust to the inclusion of site as a random effect in piecewise SEMs, which produced nearly identical path estimates and overall conclusions (Table S3).

### Top-down and bottom-up regulation of stability across trophic levels

Top-down effects clearly dominated in our model, with plants having only minor effects on herbivore stability (Fig. 2; Table S2). Specifically, we found that herbivore species diversity had the strongest effects on the adjacent trophic level. It directly promoted plant species asynchrony and plant species stability. Additionally, herbivore beta diversity negatively influenced plant community asynchrony, herbivore species stability weakly affected plant community stability, and herbivore community asynchrony and stability weakly promoted plant meta-community stability. Plant species diversity negatively affected herbivore species asynchrony. Albeit weakly, plant community stability and asynchrony destabilized and promoted herbivore meta-community stability, respectively. Stabilizing effects between adjacent trophic levels were therefore largely driven by herbivores and their diversity effects, with stability and asynchrony clearly playing inferior roles.

**Fig. 3:**
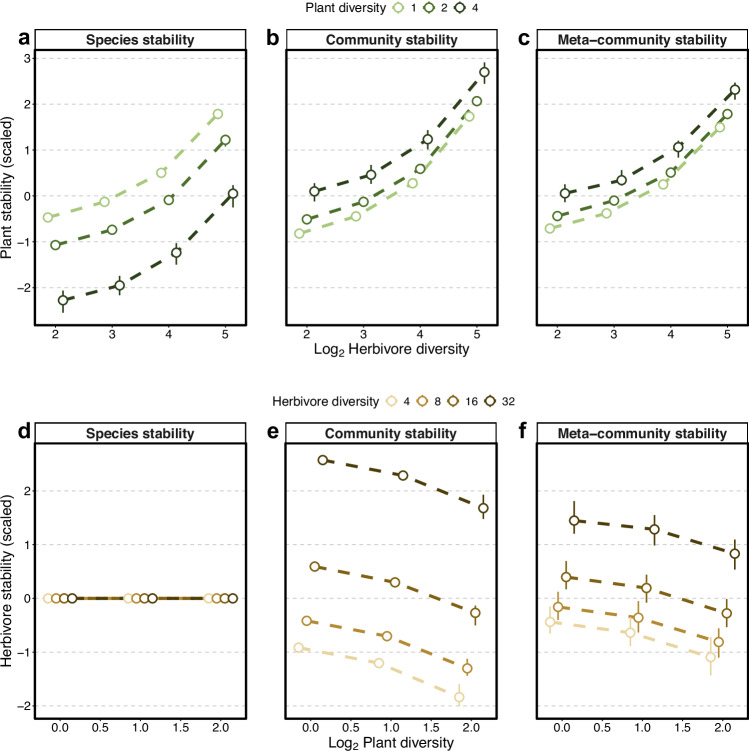
Summary of the interactive effects of plant and herbivore species diversity on stability across levels of organization from a structural equation model. Relationships are shown for plant **a** species, **b** community, and **c** meta-community stability, as well as herbivore **d** species, **e** community, and **f** meta-community stability. Values are simulated from path coefficients and standard errors of the fitted structural equation model (Fig. 2), simultaneously taking into account direct and indirect effects, with circles representing mean estimates and error bars representing the 25th–75th percentiles of simulated estimates. Points and error bars were derived from *n* = 100 simulated replicates for each combination of plant and herbivore diversity levels (see the “Methods” section for more details). Source data are provided as a Source Data file.

To better understand cascading effects on plant and herbivore stability, we summarized the results from our structural equation models at fixed levels of plant and herbivore diversity (Fig. 3). Herbivore species diversity generally promoted the stability of the adjacent trophic level (Fig. 3a–c), whereas plant species diversity showed weak negative effects on herbivore stability (Fig. 3d–f). Diversity effects within trophic levels enhanced the positive effects across trophic levels for community and meta-community stability, but not for species stability, where diversity effects were neutral (herbivore species stability; Fig. 3d) or even negative (plant species stability; Fig. 3a).

## Discussion

Our study provides insights into how the stability of populations, communities and meta-communities is linked within and between plant and herbivore trophic levels. We demonstrate that top-down processes dominate, with herbivore species diversity being the main pathway by which the plants are affected. Positive effects dominate in this context and consequently stabilize plant populations, communities, and meta-communities. Effects within trophic levels are largely stabilizing and regulated via stability metrics and asynchrony. As a result, plant stability and asynchrony emerged as mediators linking herbivore species diversity to the stability of plants at higher levels of organization. This approach thus helps to disentangle the processes that govern ecological stability within and across trophic levels, providing a means to better understand the functioning and temporal dynamics of multi-trophic communities across levels of organization.

As expected, we observed bottom-up and top-down control acting in concert in the plant-herbivore system. Interestingly, however, we found the effects of top-down regulation to be much more pronounced than bottom-up processes, with bottom-up processes being weakly negative only for community and meta-community stability. This result is particularly surprising given that our study is based on experimentally manipulated plant communities, which would be expected to lead to strong bottom-up effects. However, top-down effects may establish relatively quickly even under such conditions because, in contrast to plant species, herbivores are more flexible in utilizing resources by shifting hosts, whereas plants need to cope with the pressure from herbivores^47^. This pattern likely reflects the inherent life-history differences between the two trophic groups in our system: trees are long-lived and slow-growing, whereas herbivore populations can turn over annually and respond rapidly to environmental fluctuations. Such temporal differences in life history patterns are therefore more likely to amplify bottom-up sensitivity of herbivores to plant dynamics, as herbivore communities can adjust more quickly to short-term variations in plant growth than vice versa. In systems with contrasting life-history pairings between trophic levels, the direction and strength of cross-level regulation may change. For example, when plants are short-lived (e.g., in annual grasslands) but consumers are relatively slow to change (e.g., large mammalian grazers with multi-year life cycles), plant dynamics can respond rapidly to environmental variation and thus exert strong bottom-up control over consumer stability^8,48–50^. The dynamic advantage of insect herbivores observed in our study is likely further enhanced due to the dominance of generalist herbivores in our study region^51^, which enables a more flexible host choice. Our results are in line with expectations, suggesting that top-down regulation is associated with more immediate feedback loops in ecological systems^52^, and with observational findings suggesting that top-down effects can develop quickly and play a key role in regulating ecosystem functioning despite the direct manipulation of tree diversity^53^. The weak negative effects of trees on herbivore stability were mainly mediated by a reduction in herbivore species asynchrony with increasing tree species richness. Increased tree diversity—by expanding variation in host traits—appeared to synchronize specialist herbivores (which are limited in their ability to exploit multiple host species) more than generalists^54^, thereby reducing herbivore community and metacommunity stability.

Species diversity played a prominent role in the stability at all levels of organization and for top-down regulation. Positive effects of diversity on stability are a widely recognized phenomenon^7,55–57^. The insurance hypothesis posits that a diverse community is more likely to buffer environmental fluctuations and thereby stabilizes ecosystem functioning in time^1^. Additionally, more diverse plant communities can also buffer climate variability by creating more stable micro-climates^58,59^. Similarly, the effects of diversity through top-down regulation between trophic levels in our study were overall stabilizing, and especially driven by positive effects of herbivore species diversity (i.e., potential top-down effects of herbivore species diversity on plant species asynchrony and species stability, respectively). Our findings thus indicate that the stabilizing effects of diversity are not constrained within trophic levels but can stabilize the adjacent trophic level as well.

In addition to the positive effects of herbivore species diversity on plant stability, our analyses also showed that diversity effects were not always positive and were additionally supplemented by weak effects of asynchrony and stability. For example, herbivore beta diversity was negatively related to plant community asynchrony, likely due to the large proportion of generalist herbivores in our system^51,54^, which potentially adds a negative top-down component to the otherwise positive effects of plant community asynchrony on plant meta-community stability. This may be rooted in the fact that herbivores are much more flexible than plants in adapting to changing conditions, and hence can quickly form communities that are adapted to local conditions, dictating the dynamics of plant communities at larger levels of organization at a landscape scale^60,61^. Although our focus was on organizational scales, spatial processes are inherently involved, particularly at the metacommunity level. Spatial variation in diversity and asynchrony across local communities may further enhance stability at broader scales^62^, highlighting the need to consider spatial dynamics in future work. Additionally, microclimatic variation and variation of nutritional traits and induced chemical defenses (e.g., phenolics and tannins) among dominant plant species could be potentially important moderators that should be considered^63,64^. For example, a previous study showed that differences in understory microclimate among tree species can alter caterpillar temporal stability, indicating that microclimate-driven variation in plant quality may modify the strength of bottom-up effects^64^. As noted by Wetzel et al.^65^, variability in plant–herbivore interactions is often shaped by species identity, feeding specialization, and plant traits such as nutrient content and induced defenses, which can modulate the strength and temporal dynamics of top-down and bottom-up effects. In our system, dominant tree species and the predominance of generalist Lepidoptera herbivores likely underlie the observed stabilizing top-down effects, highlighting how specific species’ traits and interactions can influence stability patterns across organizational scales. The potentially destabilizing effects of adapting herbivores may also be the reason for the negative effects of their species asynchrony on plant community stability. Explicitly modeling top-down and bottom-up effects on stability, therefore, helps to contextualize the largely positive effects on stability across scales observed within trophic levels of plants^15,20^ and herbivores^12^, revealing that mechanisms within and across trophic levels act in parallel and potentially neutralize each other.

Despite multiple pathways by which stability is promoted across trophic levels, species diversity clearly emerged as the main driver of top-down effects. This indicates that the dynamics of herbivores and plants, captured by their stability and asynchrony metrics, are only weakly linked directly, contrasting the common association of predator-prey dynamics with an arms race that can destabilize their dynamics^66^. The relatively weak dynamic link between plant-herbivore interactions could be attributed to the dominance of generalist herbivores in our experiment^51^, and may emerge differently in other ecosystems, such as many tropical forests, where high degrees of specialization are common^67–69^. As a result, we found that the scaling of stability across levels of organization was largely constrained within trophic levels, with cascading top-down effects of herbivore species diversity stabilizing plants across levels of organization. The effects of biodiversity loss on stability are therefore two-fold. First, a reduced species diversity will diminish stability within trophic levels. Second, diversity loss can additionally have negative effects on the stability of the adjacent trophic level. Our findings suggest that these effects can amplify each other, especially for plant community and meta-community stability. However, given that our experimental system is limited to two sites in Chinese subtropical forests, it is yet unknown whether our findings are consistent across ecosystems and in more extensive landscapes. Our analytical framework can serve as an important tool to tackle these questions, and can thus be a valuable contribution to dealing with some of the challenges posed by global biodiversity change.

The scaling of stability differed between plants and herbivores, with asynchronous dynamics playing a more important role for herbivores than for plants, likely due to the herbivores’ higher adaptability to environmental shifts^9,12,69–71^. Consequently, despite being often seen as a consequence of plant diversity^27,72^, herbivore species diversity stabilizes plant communities and meta-communities primarily via plant species-level stability, while its effects on plant asynchrony are comparatively smaller. For herbivores, however, species asynchrony has stronger cascading effects. Overall, herbivore diversity not only enhances stability within its own trophic level but also appears to contribute to stabilizing plant dynamics across levels of organization.

Our analytical approach helps to extend our understanding of the effects of diversity on stability across levels of organization, from a single to adjacent trophic levels. We show that populations, communities and meta-communities within and between trophic levels are strongly driven by diversity effects. In particular, even though top-down and bottom-up processes act in concert, herbivore species diversity played a much more prominent role in stabilizing both plant and herbivore dynamics. Given the widespread decline of insect species^73^ and top-down deconstruction of food webs^74,75^, the importance of top-down processes in stabilizing plant dynamics is therefore rendered particularly sensitive to global biodiversity change. In light of this, our results underscore the importance of considering the nuanced effects of diversity on stability across trophic levels, highlighting that taking into account the multi-layered interactions between trophic levels can be key to better understand the components and scaling of stability and their consequences for ecosystem functioning.

## Methods

### Study site

We conducted our field work in the BEF-China tree biodiversity experiment^46^, located in Jiangxi Province, southeast China (29°08′–29°11′N, 117°90′–117°93′E). The mean annual temperature is 16.7 °C, and the mean annual precipitation is 1800 mm^76^. The experiment includes two study sites (A and B), which were established in 2009 and 2010, respectively. In total, 566 plots (25.8 × 25.8 m^2^) were established. On each plot, 400 saplings were planted on a regular grid with 20 rows and columns (planting distance 1.29 m).

For our study, we selected a set of 64 intensively studied plots (32 for each site, randomly distributed across the sites). The selected plots span a tree diversity gradient from monocultures to 24 species-mixtures (16 monocultures, and eight, four, two, one, and one mixtures of 2, 4, 8, 16 and 24 species, respectively, per study site). The tree species composition is largely non-overlapping between the two study sites. Only the 24-species mixtures had an overlap of eight tree species between both sites. We excluded 12 plots due to high tree mortality, resulting in a final set of 52 plots included in our analyses.

### Herbivore data

We focused on lepidopteran caterpillars, a key group of insect herbivores in forests^68,77^ that is often responsible for more than 50% of total leaf damage^78,79^. Lepidopteran herbivores were collected from 2017 to 2022 for a total of 6 years (three times per year: April, June and September). For our analysis, we pooled samples per year. We beat individual tree branches with a padded stick above a white sheet (1.5 m  × 1.5 m) and collected the fallen caterpillars^27,80^. Starting from the first row of each plot, collection was continued until 80 living trees were sampled. Caterpillars were recorded at the tree individual level and stored in separate tubes filled with 99.5% ethanol. All samples were kept in a –20 °C freezer until further processing. A molecular marker of caterpillars, the mitochondrial cytochrome c oxidase subunit I (COI), was sequenced for species delimitation. We followed Wang et al.^27^ for DNA extraction, amplification, sequencing and sequence alignment. Three methods were used to infer MOTUs: (1) threshold-based hierarchical clustering with BLASTclust, (2) Automatic Barcode Gap Discovery (ABGD), and (3) Poisson Tree Processes model (PTP). We compared the Hubert and Arabie adjusted Rand index^81^, and selected hierarchical clustering results for further analyses, as this was the most consistent delimitation method (hierarchical clustering vs. ABGD: 0.974; hierarchical clustering vs. PTP: 0.992; ABGD vs. PTP: 0.966). From the 17,850 sampled individuals, this yielded 870 MOTUs. To exclude potential bias from rare species, species represented by fewer than five individuals were excluded, leaving a total of 243 MOTUs, accounting for 14,006 individuals (Table S1).

### Tree data

Between 2016 and 2022, we measured tree height, *H*_*i*_, and basal radius, BR_*i*_, both recorded in meters, for individual trees *i* in the central 36 planting positions of a plot. We then calculated aboveground wood volume *V*_*i*_ as

1 $${V}_{i}={H}_{i}\times \pi {({\rm {B{R}}}_{i})}^{2}\times f$$

with *f* being a fixed form factor of 0.5 to reflect the noncylindrical shape of trees for the young subtropical trees in our experiment^82^. For each plot and year, accumulated tree volume *V* (m^3^ ha^−1^) was calculated as the sum of wood volumes *V*_*i*_ of the living trees in the central 36 planting positions of a plot, standardized by area. Tree absolute growth rates (TGR) per plot were then calculated as

2 $${\rm {TGR}}={V}_{2}-{V}_{1}$$

where *V*_1_ and *V*_2_ represent the tree wood volumes of two consecutive years within the sampling period 2016–2021.

### Disentangling drivers of stability across scales and trophic levels

To investigate the drivers of stability across organizational scales (i.e., species, communities, meta-communities), we utilized a framework introduced by Wang and Loreau^18^. They integrated several lines of research, effectively allowing a deconstruction of stability. Specifically, they adopted a concept originally developed to investigate diversity across spatial scales, where meta-community diversity (i.e., Gamma diversity) was partitioned into its within- and between-community components (i.e., Alpha and Beta diversity^83^). Wang and Loreau^18^ extended this idea and partitioned meta-community stability multiplicatively into community stability and community asynchrony. Additionally, community stability can be deconstructed into species stability and species asynchrony^84^. By including potential effects of diversity on stability and asynchrony following previous studies, e.g., refs. ^19–21^, we can formulate a baseline hypothesis for within-trophic level effects (Fig. S1). Although species asynchrony can covary with richness due to their mathematical formulation^85^, we interpret the asynchrony pathway as reflecting ecological compensatory dynamics among species rather than a purely statistical effect. This interpretation is supported by theoretical and empirical work showing that compensatory fluctuations and temporal covariances among species can mediate biodiversity–stability relationships independently of richness effects^9,18,21,86–88^. Building on this, we assume that relationships within trophic levels also apply across trophic levels (e.g., plant community stability affects herbivore meta-community stability; Fig. S1). A part of the proposed model is based on partially deterministic relationships (e.g., stability–asynchrony relationships within trophic level are always positive^18^). Other aspects have expected relationships based on previous work using similar frameworks (i.e., positive effects of diversity on stability within trophic levels^57^). Applying the stability framework across trophic levels additionally provides the opportunity to assess direct and cascading top-down and bottom-up effects across levels of organization. It is important to note that, while bottom-up effects from plant diversity are experimentally verified, pathways from herbivores to plants are modeled as potentially causal, based on the stability–diversity framework and supported by observational data, but lack experimental proof. Future studies involving direct manipulation of herbivore communities would be valuable to experimentally test these top-down effects, although such manipulations may be logistically challenging^89^. Nevertheless, our comprehensive approach enables a more nuanced understanding of ecological interactions and their stability, providing valuable insights for biodiversity conservation and ecosystem management.

To be able to investigate the relationships between diversity, asynchrony, and stability across organizational scales within (tree or herbivore) and across trophic levels (bottom-up and top-down), we used a resampling approach^15^ where single meta-communities were assembled from five randomly selected plots *k*, each representing a single community. In total, we considered 1000 resampled meta-communities (500 for each study site). We then calculated stability, asynchrony, and diversity for herbivores and trees based on herbivore abundances and tree growth rates, respectively.

For communities and meta-communities, we calculated abundance-weighted species diversity for both tree and herbivore communities. This has the advantage of bringing together species number and the evenness of species abundance to explain the dynamics of stability^19,90^. Therefore, at the local community level, we calculated Simpson diversity as the inverse of the Simpson index as $1/{\sum }_{i}{p}_{i}^{2}$, where *p*_*i*_ is the observed relative abundance or tree growth of herbivore or tree species *i*, respectively. We calculated Simpson diversity at community (i.e., species diversity) and meta-community levels, and obtained beta diversity (i.e., compositional turnover between communities) as the ratio of meta-community diversity to species diversity^83^.

We partitioned stability indices for both herbivores and trees at the species, community and meta-community level. Stability was calculated as the weighted average temporal invariability of herbivore abundances and tree growth rates within a meta-community^20,91^:

3 $${{\rm {species}}}\,{{\rm {stability}}}=\frac{{\sum}_{i,k}{\mu }_{i,k}}{{\sum}_{i,k}\sqrt{{w}_{{ii},{kk}}}},$$

4 $${{\rm {community}}}\,{{\rm {stability}}}=\frac{{\sum}_{k}{\mu }_{k}}{{\sum}_{k}\sqrt{{v}_{{kk}}}},$$

5 $${{\rm {meta}}}-{{\rm {community}}}\,{{\rm {stability}}}=\frac{{\sum}_{k}{\mu }_{k}}{\sqrt{{\sum}_{{kl}}{v}_{{kl}}}},$$

with ${\mu }_{i,k}$ being the mean of species *i* in subplot *k*, *μ*_*k*_ the mean of community in subplot *k*, *v*_*kk*_ the temporal variance of community in subplot *k*, *v*_*kl*_ the temporal covariance of community in subplot *k* and community in subplot *l*, and *w*_*ii,kk*_ the temporal variance of species *i* in subplot *k*

We subsequently calculated species and community asynchrony as

6 $${{\rm {species}}}\,{{\rm {asynchrony}}}=\frac{{\sum}_{i,k}\sqrt{{w}_{{ii},{kk}}}}{{\sum}_{k}\sqrt{{\sum}_{{ij}}{w}_{{ij},{kk}}}},$$

7 $${{\rm {community}}}\,{{\rm {asynchrony}}}=\frac{{\sum}_{k}\sqrt{{v}_{{kk}}}}{\sqrt{{\sum}_{{kl}}{v}_{{kl}}}},$$

with *w*_*ij,kl*_ being the temporal covariance of species *i* in subplot *k* and species *j* in subplot *l*. Note that asynchrony, as defined here, can vary with species richness when species vary independently^85^.

### Statistical analyses

All analyses were conducted in R 4.2.2 (www.r-project.org). To test whether tree and herbivore stability is top-down or bottom-up controlled, we fitted structural equation models (SEMs) using R package ‘lavaan’^92^ based on the proposed relationships between diversity, asynchrony and stability (Fig. S1). Because plots were nested within two experimental sites, we also evaluated whether including site as a random factor affected our conclusions. We first tested piecewise SEMs^93^ with site as a random effect for the model, but this approach led to singular fits because site explained negligible variance. Given that the site has only two levels, which is generally insufficient for reliable random-effects estimation^94^, we retained the simpler SEM structure in lavaan. Importantly, both approaches yielded qualitatively identical results. To optimize the model structure, we first sequentially removed non-significant pathways if their removal resulted in increased model fit (lower or equal AIC and improved fit statistics, i.e., lower SRMR, RMSEA and higher CFI). Based on modification indices, we then considered covariances between variables that we did not include in our initial model, accounting for associations through variables not included in our study (e.g., interactions with higher trophic levels). Potentially meaningful covariances were added until fit statistics were acceptable (i.e., SRMR < 0.08, RMSEA < 0.08, CFI > 0.9). We did not consider the p-value of the model due to its sensitivity to sample size, which is easily manipulated in resampling-based approaches such as ours^95,96^. When added covariances altered the significance of pathways, we reevaluated the contribution of these pathways to model fit. All relationships were defined in a unidirectional manner, from diversity to asynchrony and stability within and across trophic levels, without any reciprocal or feedback paths among variables.

To investigate the effects of plant and herbivore diversity on stability across organizational levels while taking direct and indirect pathways in the model structure into account, we simulated data at fixed diversity levels based on standardized estimates (std.est.) and standard errors (SE) of the fitted path coefficients. For each simulation, we randomly drew parameter values from normal distributions, *N* (std.est., SE), generating 100 replicates for each possible combination of the selected levels of plant and herbivore diversity (i.e., 1, 2, 4 for plants; 4, 8, 16, 32 for herbivores). This approach allowed us to visualize how plant and herbivore diversity jointly influence stability across levels of organization, accounting for uncertainty in model parameters.

### Reporting summary

Further information on research design is available in the Nature Portfolio Reporting Summary linked to this article.

## Supplementary information

Supplementary Information

Reporting Summary

Transparent Peer Review file

## Source data

Source Data

## Data Availability

The tree and herbivore data generated in this study have been deposited in the figshare^97^ at 10.6084/m9.figshare.32196165, in the Science Data Bank^98^ at 10.57760/sciencedb.36263, and in the BEF-China repository at https://data.botanik.uni-halle.de/bef-china/datasets/769. The COI sequences generated in this study have been deposited in The Genome Sequence Archive (GSA) under project PRJCA052105 (accession ID: CRA034950). Source data are provided with this paper.

## Code availability

Code is available on the Science Data Bank^98^ at 10.57760/sciencedb.36263.
