# Supplementary material for: Scaling biodiversity-stability relationships from populations to meta-communities across trophic levels
Source: Nat Commun. 2026 Jul 7;17:6038. doi: 10.1038/s41467-026-75366-1 (PMC13350715; doi:10.1038/s41467-026-75366-1)
Supplement: Supplementary file 1 — Supplementary Information [file 41467_2026_75366_MOESM1_ESM.pdf]

Scaling biodiversity-stability relationships from populations to meta-communities across trophic levels

Supplementary Information

Wang et al.

Table of contents:
Supplementary figure
    Figure 1
Supplementary tables
    Tables 1-3

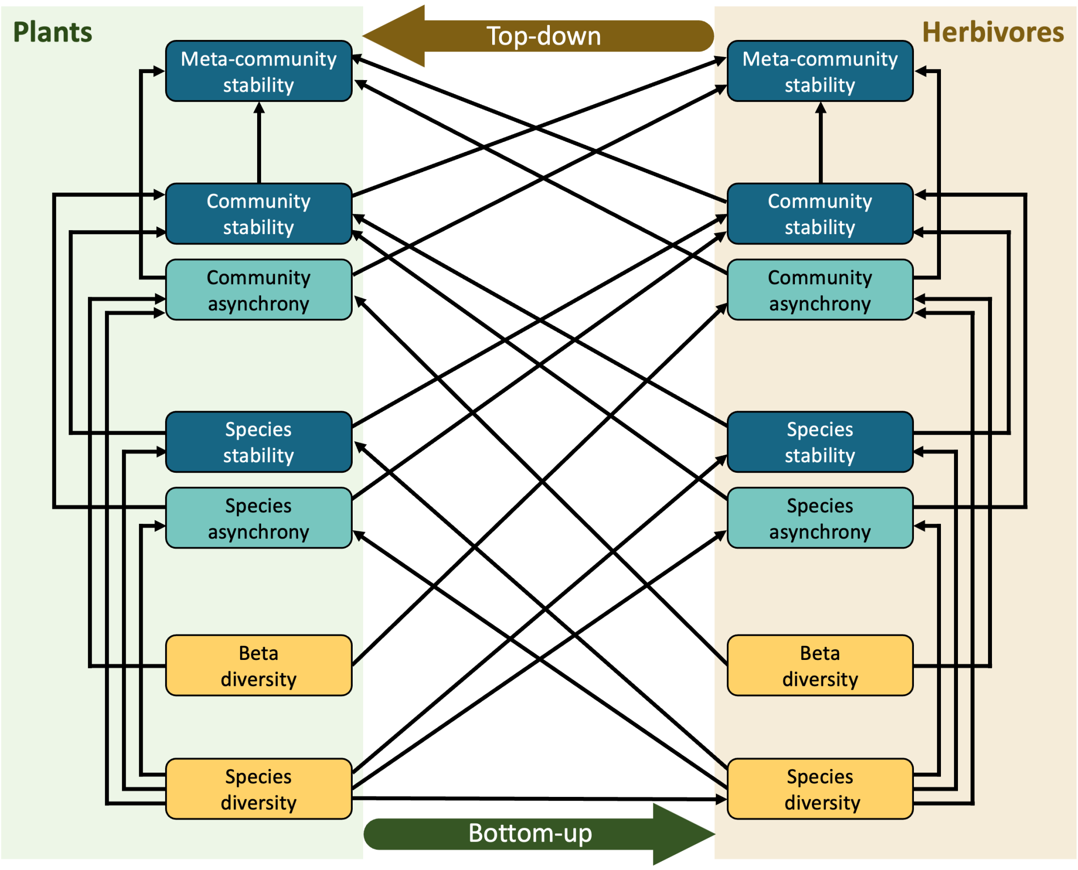

Fig. S1 Initial model structure of the path models. Structure based on theoretical expectations and correlations among herbivore- (right panel) and plant-based (left panel) variables: species diversity, species asynchrony, species stability, beta diversity, spatial asynchrony, community stability, and meta-community stability.

Table S1 Summary identification of results for Lepidoptera herbivore at the family level.

Family	MOTU	Individual
Unidentified	111	5023
Erebidae	40	3413
Geometridae	37	4294
Noctuidae	10	180
Notodontidae	7	232
Psychidae	6	130
Tortricidae	6	144
Nolidae	5	197
Limacodidae	3	15
Lycaenidae	3	22
Saturniidae	3	47
Gelechiidae	2	13
Lasiocampidae	2	194
Pyralidae	2	30
Zygaenidae	2	23
Crambidae	1	10
Drepanidae	1	25
Euteliidae	1	5
Sphingidae	1	9
Total	243	14006

Table S2. Structural equation model output for stability determining processes within and across trophic levels (with lavaan package). Statistical tests were two-sided, and no adjustments were made for multiple comparisons.

Model	
Estimator	Maximum likelihood
Number of observations	1000
CFI	0.988
RMSEA	0.070
SRMR	0.076
Chi-square	327.55
Degrees of freedom	55
P(Chi-square)	0

Regressions

	Estimate	SE	z	P	Stand. Est.
Plant meta-community stability ~					
Plant community stability	0.84	0.01	117.36	0.000	0.85
Plant community asynchrony	0.47	0.01	71.03	0.000	0.47
Herbivore community stability	0.02	0.01	2.56	0.010	0.02
Herbivore community asynchrony	0.02	0.01	2.36	0.018	0.02
Plant community stability ~		~			
Plant species stability	0.89	0.00	294.47	0.000	0.88
Plant species asynchrony	0.31	0.00	75.29	0.000	0.31
Herbivore species stability	0.01	0.00	3.15	0.002	0.01
Plant species stability ~		~			
Plant species diversity	-0.14	0.03	-5.20	0.000	-0.14
Herbivore species diversity	0.35	0.03	12.15	0.000	0.36
Plant community asynchrony ~		~			
Plant beta diversity	0.19	0.03	6.24	0.000	0.19
Herbivore beta diversity	-0.06	0.03	-1.91	0.056	-0.06
Plant species asynchrony ~		~			
Plant species diversity	0.64	0.02	29.28	0.000	0.64
Herbivore species diversity	0.29	0.02	14.01	0.000	0.29
Herbivore meta-community stability ~			~		
Herbivore community stability	0.75	0.00	273.10	0.000	0.75
Herbivore community asynchrony	0.80	0.00	299.62	0.000	0.78
Plant community stability	-0.02	0.00	-5.62	0.000	-0.02
Plant community asynchrony	0.01	0.00	5.01	0.000	0.01
Herbivore community stability ~			~		
Herbivore species stability	0.39	0.00	330.03	0.000	0.38

Herbivore species asynchrony	0.89	0.00	727.08	0.000	0.88
Herbivore community asynchrony ~					
Herbivore species diversity	-0.13	0.03	-4.98	0.000	-0.13
Herbivore beta diversity	0.48	0.03	17.96	0.000	0.49
Herbivore species asynchrony ~					
Herbivore species diversity	0.62	0.03	24.89	0.000	0.62
Plant species diversity	-0.08	0.02	-3.17	0.002	-0.08
Herbivore species diversity ~	~				
Plant species diversity	0.06	0.03	1.81	0.070	0.06

Covariances

	Estimate	SE	z	P	Stand. Est.
Herbivore species diversity ~~					
Herbivore beta diversity	-0.15	0.03	1.47	0.142	0.05
Plant species diversity ~~					
Plant beta diversity	-0.15	0.03	-5.30	0.000	-0.17
Herbivore meta-community stability~~					
Herbivore community stability	0.00	0.00	-11.33	0.000	-0.38
Herbivore species stability~~					
Herbivore beta diversity	-0.28	0.03	-8.96	0.000	-0.29
Herbivore community asynchrony ~~					
Herbivore species stability	-0.23	0.03	-9.09	0.000	-0.27
Plant Community stability~~					
Plant species asynchrony	0.03	0.00	10.16	0.000	0.44
Plant species stability~~					
Plant species asynchrony	0.16	0.02	8.45	0.000	0.25
Herbivore community asynchrony	0.23	0.02	9.66	0.000	0.29
Herbivore species asynchrony ~~					
Herbivore beta diversity	0.14	0.02	5.86	0.000	0.18
Plant meta-community stability~~					
Plant community stability	0.00	0.00	5.09	0.000	0.15
Herbivore species asynchrony~~					
Herbivore species stability	0.11	0.02	4.50	0.000	0.14
Plant species stability~~					
Herbivore beta diversity	0.19	0.03	7.31	0.000	0.21

Variances

	Estimate	SE	z	P	Stand. Est.
Plant meta-community stability	0.04	0.00	22.36	0.000	0.04
Plant community stability	0.01	0.00	19.20	0.000	0.01
Plant species stability	0.84	0.04	22.63	0.000	0.84
Plant community asynchrony	0.96	0.04	22.36	0.000	0.96
Plant species asynchrony	0.49	0.02	22.46	0.000	0.49
Plant species diversity	1.00	0.05	22.36	0.000	1.00

Plant beta diversity	1.00	0.05	22.36	0.000	1.00
Herbivore meta-community stability	0.01	0.00	22.36	0.000	0.01
Herbivore community stability	0.00	0.00	22.36	0.000	0.00
Herbivore species stability	1.00	0.04	22.43	0.000	1.00
Herbivore community asynchrony	0.72	0.03	22.51	0.000	0.72
Herbivore species asynchrony	0.62	0.03	22.36	0.000	0.62
Herbivore species diversity	1.00	0.05	22.36	0.000	1.00
Herbivore beta diversity	0.98	0.04	22.51	0.000	0.98

R-Square

	Estimate
Plant meta-community stability	0.95
Plant community stability	0.99
Plant species stability	0.14
Plant community asynchrony	0.04
Plant species asynchrony	0.51
Herbivore meta-community stability	0.99
Herbivore community stability	0.99
Herbivore community asynchrony	0.25
Herbivore species asynchrony	0.38
Herbivore species diversity	0.00

Table S3. Structural equation model output for stability determining processes within and across trophic levels (with piecewiseSEM package). Chi-Squared = 543.902 with P-value = 0 and on 46 degrees of freedom; Fisher's C = 88.298 with P-value = 0.246 and on 80 degrees of freedom. Statistical tests were two-sided, and no adjustments were made for multiple comparisons.

Response variable	Predictor	Est.	SE	DF	P	Stand. Est.
Plant meta-community staility	Plant community staility	0.85	0.01	995	0.000	0.85
Plant meta-community staility	Plant community asynchrony	0.46	0.01	995	0.000	0.46
Plant meta-community staility	Herbivore community stability	0.02	0.01	995	0.024	0.02
Plant meta-community staility	Herbivore community asynchrony	0.02	0.01	995	0.030	0.02
Plant community staility	Plant species staility	0.88	0.00	1000	0.000	0.88
Plant community staility	Plant species asynchrony	0.33	0.00	1000	0.000	0.33
Plant community staility	Herbivore species stability	0.01	0.00	1000	0.020	0.01
Plant species staility	Plant species diversity	-0.10	0.03	1000	0.000	-0.10
Plant species staility	Herbivore species diversity	0.12	0.03	1000	0.000	0.12
Plant community asynchrony	Plant beta diversity	0.21	0.03	1000	0.000	0.21
Plant community asynchrony	Herbivore beta diversity	-0.03	0.03	1000	0.398	-0.03
Plant species asynchrony	Plant species diversity	0.66	0.02	1000	0.000	0.66
Plant species asynchrony	Herbivore species diversity	0.23	0.02	1000	0.000	0.23
Herbivore meta-community stability	Herbivore community stability	0.75	0.00	995	0.000	0.75
Herbivore meta-community stability	Herbivore community asynchrony	0.80	0.00	995	0.000	0.80
Herbivore meta-community stability	Plant community staility	-0.02	0.00	995	0.000	-0.02
Herbivore meta-community stability	Plant community asynchrony	0.01	0.00	995	0.000	0.01
Herbivore community stability	Herbivore species stability	0.39	0.00	997	0.000	0.39
Herbivore community stability	Herbivore species asynchrony	0.89	0.00	997	0.000	0.89

Herbivore community asynchrony	Herbivore species diversity	-0.32	0.03	1000	0.000	-0.32
Herbivore community asynchrony	Herbivore beta diversity	0.39	0.02	1000	0.000	0.39
Herbivore species asynchrony	Herbivore species diversity	0.70	0.03	1000	0.000	0.70
Herbivore species asynchrony	Plant species diversity	-0.14	0.02	1000	0.000	-0.14
Herbivore species diversity	Plant species diversity	0.10	0.03	1000	0.001	0.10
**Covariances**						
~~Herbivore meta-community stability	~~Herbivore community stability	0.99	-	1000	0.000	0.99
~~Herbivore species stability	~~Herbivore beta diversity	-0.33	-	998	0.000	-0.33
~~Herbivore community asynchrony	~~Herbivore species stability	-0.25	-	1000	0.000	-0.25
~~Plant community staility	~~Plant species asynchrony	0.97	-	1000	0.000	0.97
~~Plant species staility	~~Plant species asynchrony	0.26	-	1000	0.000	0.26
~~Plant species staility	~~Herbivore community asynchrony	0.14	-	1000	0.000	0.14
~~Herbivore species asynchrony	~~Herbivore beta diversity	0.23	-	1000	0.000	0.23
~~Plant meta-community staility	~~Plant community staility	0.97	-	1000	0.000	0.97
~~Herbivore species asynchrony	~~Herbivore species stability	0.08	-	1000	0.006	0.08
~~Plant species staility	~~Herbivore beta diversity	0.22	-	1000	0.000	0.22
~~Plant species staility	~~Herbivore species stability	-0.09	-	1000	0.003	-0.09
~~Plant species asynchrony	~~Herbivore species stability	-0.12	-	1000	0.000	-0.12
~~Herbivore species diversity	~~Herbivore species stability	0.10	-	1000	0.001	0.10
~~Plant community staility	~~Plant species diversity	-0.21	-	1000	0.000	-0.21

~~Herbivore community asynchrony	~~Plant species diversity	0.09	-	1000	0.001	0.09
~~Plant meta-community staility	~~Plant beta diversity	0.08	-	1000	0.006	0.08
~~Plant species asynchrony	~~Herbivore beta diversity	0.13	-	1000	0.000	0.13
~~Plant meta-community staility	~~Herbivore beta diversity	0.10	-	1000	0.001	0.10
~~Plant species staility	~~Plant community asynchrony	0.14	-	1000	0.000	0.14
~~Plant species asynchrony	~~Plant community asynchrony	0.07	-	1000	0.014	0.07
~~Herbivore species diversity	~~Plant community asynchrony	-0.15	-	1000	0.000	-0.15
~~Herbivore species asynchrony	~~Plant species asynchrony	-0.20	-	1000	0.000	-0.20
~~Plant community staility	~~Herbivore species diversity	-0.16	-	1000	0.000	-0.16
~~Herbivore community asynchrony	~~Herbivore species asynchrony	0.15	-	1000	0.000	0.15
~~Herbivore community asynchrony	~~Herbivore community stability	-0.08	-	1000	0.007	-0.08

R-Square

Response	Method	Marginal	Conditional
Plant meta-community staility	none	0.96	NA
Plant community staility	none	0.99	0.99
Plant species staility	none	0.03	0.32
Plant community asynchrony	none	0.04	0.06
Plant species asynchrony	none	0.5	0.51
Herbivore meta-community stability	none	0.99	NA
Herbivore community stability	none	1	NA
Herbivore community asynchrony	none	0.24	0.46
Herbivore species asynchrony	none	0.44	0.48
Herbivore species diversity	none	0.01	0.2
