# Supplementary material for: Scaling biodiversity-stability relationships from populations to meta-communities across trophic levels
Source: Nat Commun. 2026 Jul 7;17:6038. doi: 10.1038/s41467-026-75366-1 (PMC13350715; doi:10.1038/s41467-026-75366-1)
Supplement: Supplementary file 3 — Transparent Peer Review file [file 41467_2026_75366_MOESM3_ESM.pdf]

nature portfolio

Peer Review File

Scaling biodiversity-stability relationships from populations to meta-communities across trophic levels

Corresponding Author: Dr Ming-Qiang Wang

This file contains all reviewer reports in order by version, followed by all author rebuttals in order by version.

Version 0:

Reviewer comments:

Reviewer #1

(Remarks to the Author)
The manuscript by Wang et al. utilizes five years of tree growth and herbivore species abundance data from the BEF China experiment to assess how diversity within each tree and herbivore trophic group influences the stability in the growth and abundance of each at the community and meta-community level. Further they assess the top-down effects of the species diversity on the stability and asynchrony in tree communities as well as the bottom-up effects of plant community stability on the stability of the herbivore (meta)-community. The study nicely shows that the diversity stability relationships across the level of organization (species, community and meta-community) are similar for both trees and herbivores, there is a stronger top-down effect of herbivore diversity on the stability of tree community than the bottom-up effects of the trees on herbivores. This is a nice study that is well rooted in the theory and concepts and provides important insights into how diversity stability relationships transcend across trophic groups of organisms. I quite liked the simplicity of figure 3 that nicely summarizes the interactive effects of top-down and bottom up effects on the stability of tree and herbivore communities.

My general comments and thoughts on the manuscript was that it is a bit of a conceptually heavy paper in parts that I had to read through a few times to grasp what was being described due to the inherent content. However, it is a generally well written paper.
My first reaction to the results were that these findings are largely due to the nature of the specific system they are studying (trees and herbivores). Specifically, the differences in life-cycle timelines where herbivores may have an annual turnover in species populations while trees live much longer with likely no species population turnover. Thus herbivores species and populations are better able to respond more rapidly in any year to year variation in tree growth while the trees are much less able to respond rapidly to within year or year to year changes in the herbivore communities. This is largely already address in the discussion (i.e. L204 – 216) however, the discussion is very general in applying the findings to plants-herbivore interactions than discussing the limitations of the top-down driven results that may only apply to such a slow developing system. It would be nice to consider how these results may contrast with other systems, such as grasslands whether the results would be opposite if the life strategies were different between the two trophic groups (i.e. large mammals grazing in an annual plant community).

Specific lines:
L143/144. We are talking about "tree diversity" here? (not herbivore diversity)
L156-57. Is this not because species synchrony as calculated here is inherently associated with species richness within a community? (see Gross et al. 2017. American Naturalist https://doi.org/10.1086/673915)
L183: "plants species" should be "plant species"
L232 - : This makes me wonder if the herbivores are more specialist ore generalist herbivores, where I would assume that specialists might more drive plant asynchrony (different herbivores feeding on different species at different times?) while generalists affect all plant species similarly? Ok I see this is nicely answered below in the text.
L268 - : I found this paragraph to be a bit redundant repeating some statements above. Consider condensing.

Reviewer #2

(Remarks to the Author)
In their paper "Scaling biodiversity–stability relationships from populations to meta-communities across trophic levels," Wang

et al. analyse data from a large-scale forest biodiversity experiment at two sites to investigate how temporal stability scales from populations to communities and meta-communities in a plant–herbivore system. The study also explores potential top-down and bottom-up effects on biodiversity–stability relationships. The authors find that biodiversity generally has stabilizing effects within and between trophic levels, with herbivore diversity emerging as a key mediator of plant stability via asynchrony.
Overall, the manuscript is clearly written, well structured, and makes a potentially valuable contribution to biodiversity–stability theory. However, I have some concerns that need to be addressed before the paper can be considered for publication. Below, I outline my major and specific comments.

Major Comments
1. Causality and experimental design
The strongest concern lies in the causal interpretations. The experimental design manipulates plant monocultures and mixtures at two sites, with herbivore communities (lepidopteran caterpillars) subsequently sampled. This structure clearly reflects a bottom-up design, with trees as the manipulated component. Therefore, while plant-to-herbivore effects can be robustly examined, herbivore-to-plant “top-down” effects cannot be established experimentally. At best, such effects can be inferred from correlation. I recommend the authors (i) focus primarily on bottom-up causal effects or (ii) treat herbivore-to-plant relationships as associations rather than causal pathways.
Additionally, the plant data span from 2016 to 2021, whereas the herbivore data cover the period from 2017 to 2022. Because 2016 includes only plants and 2022 only herbivores, temporal mismatches reduce the strength of plant–herbivore associations. Restricting analyses to the 2017–2021 period, when both groups were sampled simultaneously, would provide a more consistent dataset.

2. Scalability of results
The experiment includes only two sites, each with 32 subplots. This raises questions about the generalizability of the observed patterns to larger or more heterogeneous systems. The authors should explicitly discuss the limits of inference and how results may or may not extend beyond the study system.

3. Statistical analysis
Given that plots are nested within sites, the analyses should account for hierarchical structure. A linear mixed model or generalized linear mixed model framework would be appropriate, and a piecewise SEM (Lefcheck 2016) could provide a flexible way to integrate multiple paths. Clarifying this would strengthen confidence in the robustness of the results.

Lefcheck, J. S. (2016). piecewiseSEM: Piecewise structural equation modelling in R for ecology, evolution, and systematics. Methods in Ecology and Evolution, 7(5), 573-579.

4. SEM model circularity
The SEM appears to include circular pathways (meaning you have a variable whose paths feed back into that variable), which can compromise model fit and interpretation. How does the lavaan framework used here address circularity? The authors should clarify whether feedback loops exist in the model and, if so, how they are handled statistically.

5. Biological interpretation
The manuscript would benefit from a stronger integration of biological detail regarding the species involved and their interactions. For example, which tree and herbivore species (or groups) were most dominant over time, and how might their dynamics have shaped the observed stability patterns? Providing more information on the specific herbivore–plant relationships at the study sites would also help contextualize the results. Interactions in such systems are often mediated by important aspects, such as nutrient heterogeneity, plant-induced defences, and herbivore generality, that could be directly relevant to interpreting the findings (Wetzel et al. 2023). Including this level of ecological specificity would strengthen the connection between the statistical results and their biological meaning.
Wetzel, William C., et al. "Variability in plant–herbivore interactions." Annual Review of Ecology, Evolution, and Systematics 54.1 (2023): 451–474.

Specific Comments
Lines 83–87: This section appears disconnected from the preceding text. Consider moving it to a later discussion on trophic interactions.
Figure 1: The caption currently mislabels stability (panel 2) and asynchrony (panel 3). Please correct this to match the figure.

Version 1:

Reviewer comments:

Reviewer #1

(Remarks to the Author)
The authors have adequately addressed all of my questions and concerns.

Reviewer #2

(Remarks to the Author)

I congratulate the authors on the significant improvements made to the manuscript following my and Reviewer 1's suggestions. The authors have addressed my comments thoroughly and provided satisfactory justifications where appropriate.

Open Access This Peer Review File is licensed under a Creative Commons Attribution 4.0 International License, which permits use, sharing, adaptation, distribution and reproduction in any medium or format, as long as you give appropriate credit to the original author(s) and the source, provide a link to the Creative Commons license, and indicate if changes were made.
In cases where reviewers are anonymous, credit should be given to 'Anonymous Referee' and the source.
The images or other third party material in this Peer Review File are included in the article's Creative Commons license, unless indicated otherwise in a credit line to the material. If material is not included in the article's Creative Commons license and your intended use is not permitted by statutory regulation or exceeds the permitted use, you will need to obtain permission directly from the copyright holder.
To view a copy of this license, visit https://creativecommons.org/licenses/by/4.0/

Nature Communications: NCOMMS- 25-52373-T

Scaling biodiversity–stability relationships from populations to meta-communities across trophic levels

Ming-Qiang Wang, Shaopeng Wang, Xiaojuan Liu, Lei Zhao, Douglas Chesters, Helge Bruelheide, Yi Li, Jing-Ting Chen, Shan Li, Qing-Song Zhou, Keping Ma, Arong Luo, Andreas Schuldt, Chao-Dong Zhu, Georg Albert

RESPONSE TO REVIEWER 1

Reviewer #1 (Remarks to the Author):

The manuscript by Wang et al. utilizes five years of tree growth and herbivore species abundance data from the BEF China experiment to assess how diversity within each tree and herbivore trophic group influences the stability in the growth and abundance of each at the community and meta-community level. Further they assess the top-down effects of the species diversity on the stability and asynchrony in tree communities as well as the bottom-up effects of plant community stability on the stability of the herbivore (meta)-community. The study nicely shows that the diversity stability relationships across the level of organization (species, community and meta-community) are similar for both trees and herbivores, there is a stronger top-down effect of herbivore diversity on the stability of tree community than the bottom-up effects of the trees on herbivores. This is a nice study that is well rooted in the theory and concepts and provides important insights into how diversity stability relationships transcend across trophic groups of organisms. I quite liked the simplicity of figure 3 that nicely summarizes the interactive effects of top-down and bottom up effects on the stability of tree and herbivore communities.

My general comments and thoughts on the manuscript was that it is a bit of a conceptually heavy paper in parts that I had to read through a few times to grasp what was being described due to the inherent content. However, it is a generally well written paper.
Response: Thank you very much for your encouraging evaluation and constructive feedback to our work. We have revised our manuscript according to all your comments to further strengthen the clarity and coherence of our study, please see below.

My first reaction to the results were that these findings are largely due to the nature of the specific system they are studying (trees and herbivores). Specifically, the differences in life-cycle timelines where herbivores may have an annual turnover in species populations while trees live much longer with likely no species population turnover. Thus herbivores species and populations are better able to respond more rapidly in any year to year variation in tree growth while the trees are much less able to respond rapidly to within year or year to year changes in the herbivore communities. This is largely already address in the discussion (i.e. L204 – 216) however, the discussion is very general in applying the findings to plants-herbivore interactions than discussing the limitations of the top-down driven results that may only apply to such a slow developing system. It would be nice to

consider how these results may contrast with other systems, such as grasslands whether the results would be opposite if the life strategies were different between the two trophic groups (i.e. large mammals grazing in an annual plant community).

Response: We thank the reviewer for this insightful comment. We fully agree that the relatively slow dynamics of long-lived trees compared to fast-turnover herbivore populations may contribute to the dominance of top-down effects observed in our system. We have explicitly further discussed this point in the Discussion (see L220-L231). Moreover, we now also discuss the expectation that in ecosystems with faster plant turnover such as annual grasslands stronger bottom-up effects might be expected (see L227-L231).

"This pattern likely reflects the inherent life-history differences between the two trophic groups in our system: trees are long-lived and slow-growing, whereas herbivore populations can turn over annually and respond rapidly to environmental fluctuations. Such temporal differences in life history patterns are therefore more likely to amplify bottom-up sensitivity of herbivores to plant dynamics, as herbivore communities can adjust more quickly to short-term variations in plant growth than vice versa. In systems with contrasting life-history pairings between trophic levels, the direction and strength of cross-level regulation may change. For example, when plants are short-lived (e.g. in annual grasslands) but consumers are relatively slow to change (e.g. large mammalian grazers with multi-year life cycles), plant dynamics can respond rapidly to environmental variation and thus exert strong bottom-up control over consumer stability."

Specific lines:

L143/144. We are talking about "tree diversity" here? (not herbivore diversity)

Response: Yes, we have now specified this to improve clarity.

L156-57. Is this not because species synchrony as calculated here is inherently associated with species richness within a community? (see Gross et al. 2017. American Naturalist https://doi.org/10.1086/673915)

Response: Thank you very much for bringing this issue to our attention. After carefully reading the study by Gross et al., we acknowledge that the authors mention that the asynchrony metric we are using tends to increase with species richness, particularly when species vary independently. However, this relationship is not guaranteed when species are strongly correlated or when species abundance distributions are highly uneven (Thibaut & Connolly 2013; Hautier et al. 2014). Furthermore, as Gross et al. noted, different synchrony metrics serve distinct research purpose. Their analysis confirmed that synchrony defined by average correlation is a valid measure for quantifying the effect of species interactions on pairwise correlations. In contrast, for the specific purpose of assessing community stability as in our study, the synchrony metric we used provides the most direct link to community temporal variability. That is, community variability can be explicitly expressed as the product of average species variability and the synchrony metric we used (Thibaut & Connolly 2013), making this metric particularly suitable for stability analyses. This is why it has been commonly adopted in related studies (Hautier et al. 2014; Xu et al. 2021; Liang et al. 2025). We therefore decided to mention the potential issue when defining asynchrony in the Methods (L457-L458), but stick with our ecological interpretation instead of treating it like a mathematical artefact.

"Note that asynchrony as defined here can vary with species richness when species vary independently[89]"

References:

Thibaut, L. M. & Connolly, S. R. Understanding diversity–stability relationships: towards a unified model of portfolio effects. Ecol. Lett. 16, 140-150 (2013).

Hautier, Y. et al. Eutrophication weakens stabilizing effects of diversity in natural grasslands. Nature 508, 521-525 (2014).

Xu, Q. et al. Consistently positive effect of species diversity on ecosystem, but not population, temporal stability. Ecol. Lett. 24, 2256-2266 (2021).

Liang, M. et al. Unifying spatial scaling laws of biodiversity and ecosystem stability. Science 387, eadl2373 (2025).

L183: "plants species" should be "plant species"

Response: We have corrected "plants species" to "plant species."

L232 - : This makes me wonder if the herbivores are more specialist ore generalist herbivores, where I would assume that specialists might more drive plant asynchrony (different herbivores feeding on different species at different times?) while generalists affect all plant species similarly? Ok I see this is nicely answered below in the text.

Response: Thank you for noting this. We now mention the dominance of generalists already here (L258-L262).

"For example, herbivore beta diversity reduced plant community asynchrony, likely due to the large proportion of generalist herbivores in our system[51], which adds a negative top-down component to the otherwise positive effects of plant community asynchrony on plant meta-community stability."

L268 - : I found this paragraph to be a bit redundant repeating some statements above. Consider condensing.

Response: Thank you for the suggestion. We have condensed the paragraph to reduce redundancy (see L310-L319).

"The scaling of stability differed between plants and herbivores, with asynchronous dynamics playing a more important role for herbivores than for plants, likely due to the herbivores' higher adaptability to environmental shifts[9,12,69-71]. Consequently, despite being often seen as a consequence of plant diversity[27,72], herbivore species diversity stabilizes plant communities and meta-communities primarily via plant species-level stability, while its effects on plant asynchrony are comparatively smaller. For herbivores, however, species asynchrony has stronger cascading effects. Overall, herbivore diversity not only enhances stability within its own trophic level but also appears to contributes to stabilizing plant dynamics across levels of organization."

RESPONSE TO REVIEWER 2

Reviewer #2 (Remarks to the Author):

In their paper “Scaling biodiversity–stability relationships from populations to meta-communities across trophic levels,” Wang et al. analyse data from a large-scale forest biodiversity experiment at two sites to investigate how temporal stability scales from populations to communities and meta-communities in a plant–herbivore system. The study also explores potential top-down and bottom-up effects on biodiversity–stability relationships. The authors find that biodiversity generally has stabilizing effects within and between trophic levels, with herbivore diversity emerging as a key mediator of plant stability via asynchrony.

Overall, the manuscript is clearly written, well structured, and makes a potentially valuable contribution to biodiversity–stability theory. However, I have some concerns that need to be addressed before the paper can be considered for publication. Below, I outline my major and specific comments.

Response: Thank you very much for your positive assessment of our manuscript. We have carefully considered all comments and suggestions and provide detailed responses and corresponding revisions below.

Major Comments

1. Causality and experimental design

The strongest concern lies in the causal interpretations. The experimental design manipulates plant monocultures and mixtures at two sites, with herbivore communities (lepidopteran caterpillars) subsequently sampled. This structure clearly reflects a bottom-up design, with trees as the manipulated component. Therefore, while plant-to-herbivore effects can be robustly examined, herbivore-to-plant “top-down” effects cannot be established experimentally. At best, such effects can be inferred from correlation. I recommend the authors (i) focus primarily on bottom-up causal effects or (ii) treat herbivore-to-plant relationships as associations rather than causal pathways.

Additionally, the plant data span from 2016 to 2021, whereas the herbivore data cover the period from 2017 to 2022. Because 2016 includes only plants and 2022 only herbivores, temporal mismatches reduce the strength of plant–herbivore associations. Restricting analyses to the 2017–2021 period, when both groups were sampled simultaneously, would provide a more consistent dataset.

Response: We fully acknowledge that herbivores were not experimentally manipulated, and thus the top-down pathways from herbivores to plants cannot be considered strictly experimentally verified. However, within the stability–diversity framework, these pathways represent hypothesized cross-trophic interactions that we aimed to evaluate, rather than established theoretical expectations. Their inclusion in the SEM is therefore justified as part of our a priori model structure designed to test the potential magnitude and direction of such top-down associations. This approach allows us to quantify the relative importance of potential top-down effects compared to bottom-up effects, even if experimental manipulation is lacking. We now clarified this in the manuscript (see L415-L419). Moreover, we emphasized that future studies should consider manipulating herbivore communities to highlight the importance of testing top-down effects to the reader (see L419-L421).

"It is important to note that, while bottom-up effects from plant diversity are experimentally verified, pathways from herbivores to plants are modeled as potentially causal, based on the stability–diversity framework and supported by observational data, but lack experimental verification."

"Future studies involving direct manipulation of herbivore communities would be valuable to experimentally test these top-down effects, although such manipulations may be logistically challenging[89]."

To address the temporal mismatch, we have now included tree growth data from 2016 to 2022, ensuring complete overlap with herbivore data from 2017 to 2022. Please note that the 2016 tree data are included solely to calculate tree growth in 2017 (i.e., the difference between 2017 and 2016), and are not otherwise used for stability analyses. This adjustment strengthens the consistency of cross-trophic comparisons and allows more robust assessment of stability pathways across years. Now we used the fully matched dataset, which ensures improved temporal alignment and more complete capture of interannual variation. We observe that the new results remain largely consistent: herbivore diversity continues to have strong stabilizing effects on plants, while plant diversity shows only weak destabilizing effects on herbivore stability (see L181-L184; L191-193).

"…herbivore species stability weakly affected plant community stability, and herbivore community asynchrony and stability weakly promoted plant meta-community stability. Plant species diversity negatively affected herbivore species asynchrony."

"Herbivore species diversity generally promoted the stability of the adjacent trophic level (Fig.3a-c), whereas plant species diversity showed weak negative effects on herbivore stability (Fig. 3d-f)."

2. Scalability of results

The experiment includes only two sites, each with 32 subplots. This raises questions about the generalizability of the observed patterns to larger or more heterogeneous systems. The authors should explicitly discuss the limits of inference and how results may or may not extend beyond the study system.

Response: Thank you! While we would like to emphasize that in the context of biodiversity experiments our study is based on an unprecedented dataset in terms of replication and temporal scale, we were aware of this issue, hence mentioned it in lines 307, but now expanded the section to make this point more clear (see L304-L307).

"However, given that our experimental system is limited to two sites in Chinese subtropical forests, it is yet unknown whether our findings are consistent across ecosystems and in more extensive landscapes."

3. Statistical analysis

Given that plots are nested within sites, the analyses should account for hierarchical structure. A

linear mixed model or generalized linear mixed model framework would be appropriate, and a piecewise SEM (Lefcheck 2016) could provide a flexible way to integrate multiple paths. Clarifying this would strengthen confidence in the robustness of the results.

Lefcheck, J. S. (2016). piecewiseSEM: Piecewise structural equation modelling in R for ecology, evolution, and systematics. Methods in Ecology and Evolution, 7(5), 573-579.

Response: Thank you very much for this valuable suggestion. We agree that hierarchical structure is an important consideration. However, since our study includes only two sites, treating "site" as a random effect is not statistically recommended (factors with fewer than five levels generally yield unreliable variance estimates; see https://bbolker.github.io/mixedmodels-misc/glmmFAQ.html#should-i-treat-factor-xxx-as-fixed-or-random). We nevertheless tested this approach, but ran into singularity issues, indicating that site explained little variance (see L463-L470 in Methods).

Importantly, however, model estimates and qualitative conclusions were nearly identical with and without site included as a random effect, and also consistent with those obtained from the SEM implemented in lavaan. For transparency, we now report the piecewise SEM (where we addressed the singularity issues by dropping site as a random factor from those sub-models with singularity issues) in the Appendix and note that additional covariances were added to satisfy the d-separation criterion (Fisher's C > 0.05). In the main text, we clarify that accounting for site did not alter model outcomes qualitatively, thus supporting the robustness of our conclusions (see L173-L175 in Results).

In methods: "Because plots were nested within two experimental sites, we also evaluated whether including site as a random factor affected our conclusions. We first tested piecewise SEMs with site as a random effect for the model, but this approach led to singular fits because site explained negligible variance. Given that site has only two levels, which is generally insufficient for reliable random-effects estimation, we retained the simpler SEM structure in lavaan. Importantly, both approaches yielded qualitatively identical results."

In results: "Model results were robust to the inclusion of site as a random effect in piecewise SEMs, which produced nearly identical path estimates and overall conclusions (Table S3)."

4. SEM model circularity

The SEM appears to include circular pathways (meaning you have a variable whose paths feed back into that variable), which can compromise model fit and interpretation. How does the lavaan framework used here address circularity? The authors should clarify whether feedback loops exist in the model and, if so, how they are handled statistically.

Response: There are no circular pathways in our SEMs. All relationships were specified in a unidirectional manner, from lower to higher organizational levels and between trophic levels, following our conceptual model (Fig. S1). No variable feeds back into itself, either directly or indirectly. We have now clarified this explicitly in the Methods section (see L480-L483).

"All relationships were defined in a unidirectional manner, from diversity to asynchrony and

stability within and across trophic levels, without any reciprocal or feedback paths among variables."

5. Biological interpretation

The manuscript would benefit from a stronger integration of biological detail regarding the species involved and their interactions. For example, which tree and herbivore species (or groups) were most dominant over time, and how might their dynamics have shaped the observed stability patterns? Providing more information on the specific herbivore–plant relationships at the study sites would also help contextualize the results. Interactions in such systems are often mediated by important aspects, such as nutrient heterogeneity, plant-induced defences, and herbivore generality, that could be directly relevant to interpreting the findings (Wetzel et al. 2023). Including this level of ecological specificity would strengthen the connection between the statistical results and their biological meaning.

Wetzel, William C., et al. "Variability in plant–herbivore interactions." Annual Review of Ecology, Evolution, and Systematics 54.1 (2023): 451–474.

Response: Thanks for the comment. Following your suggestion, we have strengthened the biological interpretation throughout the Discussion by integrating more ecological details about the dominant species and their interactions, in addition to the sections that discussed the potential role of host specialization of the herbivore communities in our study system (see L-L; L270-L282). Furthermore, as recommended, we now provide a concise summary of the herbivore community composition to offer clearer biological context for the cross-trophic pathways (see L149-L157).

"Additionally, microclimatic variation and variation of nutritional traits and induced chemical defenses (e.g. phenolics and tannins) among dominant plant species could be potentially important moderators that should be considered[63,64]. For example, a previous study showed that differences in understory microclimate among tree species can alter caterpillar temporal stability, indicating that microclimate-driven variation in plant quality may modify the strength of bottom-up effects[64]. As noted by Wetzel et al.[65], variability in plant–herbivore interactions is often shaped by species identity, feeding specialization, and plant traits such as nutrient content and induced defenses, which can modulate the strength and temporal dynamics of top-down and bottom-up effects. In our system, dominant tree species and the predominance of generalist Lepidoptera herbivores likely underlie the observed stabilizing top-down effects, highlighting how specific species' traits and interactions can influence stability patterns across organizational scales."

"Of the 243 MOTUs included in the final analysis, 132 could be assigned to Lepidoptera families. These family-level identifiable MOTUs were dominated by several species-rich and abundant groups (Table S1), particularly Erebidae (n = 40 MOTUs), Geometridae (n = 37), and Noctuidae (n = 10), which together contributed the largest share of both richness (n = 87) and individuals (n = 7887). On average, MOTUs exhibited moderately broad generality (mean host breadth = 9.42), indicating that broad-feeding taxa were prevalent in this system. Most abundant MOTUs occurred repeatedly across multiple years, suggesting the herbivore community structured by persistent generalists rather than narrowly specialized taxa. This overall community composition provides essential biological context for interpreting the cross-trophic diversity–stability pathways observed in our study."

Specific Comments

Lines 83–87: This section appears disconnected from the preceding text. Consider moving it to a later discussion on trophic interactions.

Response: Thanks for the suggestion. Now the section has been removed to promote logic connection of the text.

Figure 1: The caption currently mislabels stability (panel 2) and asynchrony (panel 3). Please correct this to match the figure.

Response: The figure now has been corrected to match the caption.
